# Renal primitive neuroectodermal tumor: A rare case with a good prognosis

**DOI:** 10.3389/fsurg.2023.1180107

**Published:** 2023-04-19

**Authors:** Hanmin Chen, Yanmin Li, Qingming Zeng, Gengqing Wu

**Affiliations:** Department of Urology, The First Affiliated Hospital of Gannan Medical University, Ganzhou, China

**Keywords:** renal primitive neuroectodermal tumor, radical surgery, chemoradiotherapy, rare case, good survival prognosis

## Abstract

**Background:**

Renal primitive neuroectodermal tumor (rPNET) has the characteristics of a difficult preoperative diagnosis, a high degree of malignancy, easy early metastasis or postoperative recurrence, a poor prognosis, and so on. However, rPNET that has no metastasis before surgery can have a good survival prognosis only after radical surgical resection.

**Methods:**

We report the case of a 14-year-old male patient with a renal tumor who underwent open radical left nephrectomy without radiotherapy or chemotherapy before or after surgery, as confirmed by postoperative pathological results. The prognosis was followed up by a regular review of the chest and whole abdomen on CT, hematuria analysis, renal function, and electrolytes according to the guidelines for renal cancer.

**Results:**

Postoperative pathological results confirmed rPNET; no adjuvant radiotherapy or chemotherapy were performed after surgery; no tumor recurrence or metastasis were observed during the follow-up of nearly 5 years.

**Conclusions:**

Despite the high degree of rPNET malignancy, patients without metastases before surgery can still obtain a good survival prognosis through timely radical surgery.

## Introduction

Primitive neuroectodermal tumor (PNET) is a highly malignant small round-cell tumor with neurogenic differentiation (1). With the improvement of diagnostic techniques, its incidence has increased, mainly in children and adolescents, regardless of gender. PNET usually occurs in the trunk, bones, or soft tissue (2) but is rare in the kidney; less than 200 cases have been reported in the literature. rPNET is more prone to recurrence and metastasis than other renal malignancies, and the prognosis is poor, but rPNET without metastasis before surgery can achieve a good survival prognosis with timely radical intervention. We report a case of rPNET pathologically diagnosed after radical surgical resection with a good survival prognosis.

## Case presentation

**Figure 1:**
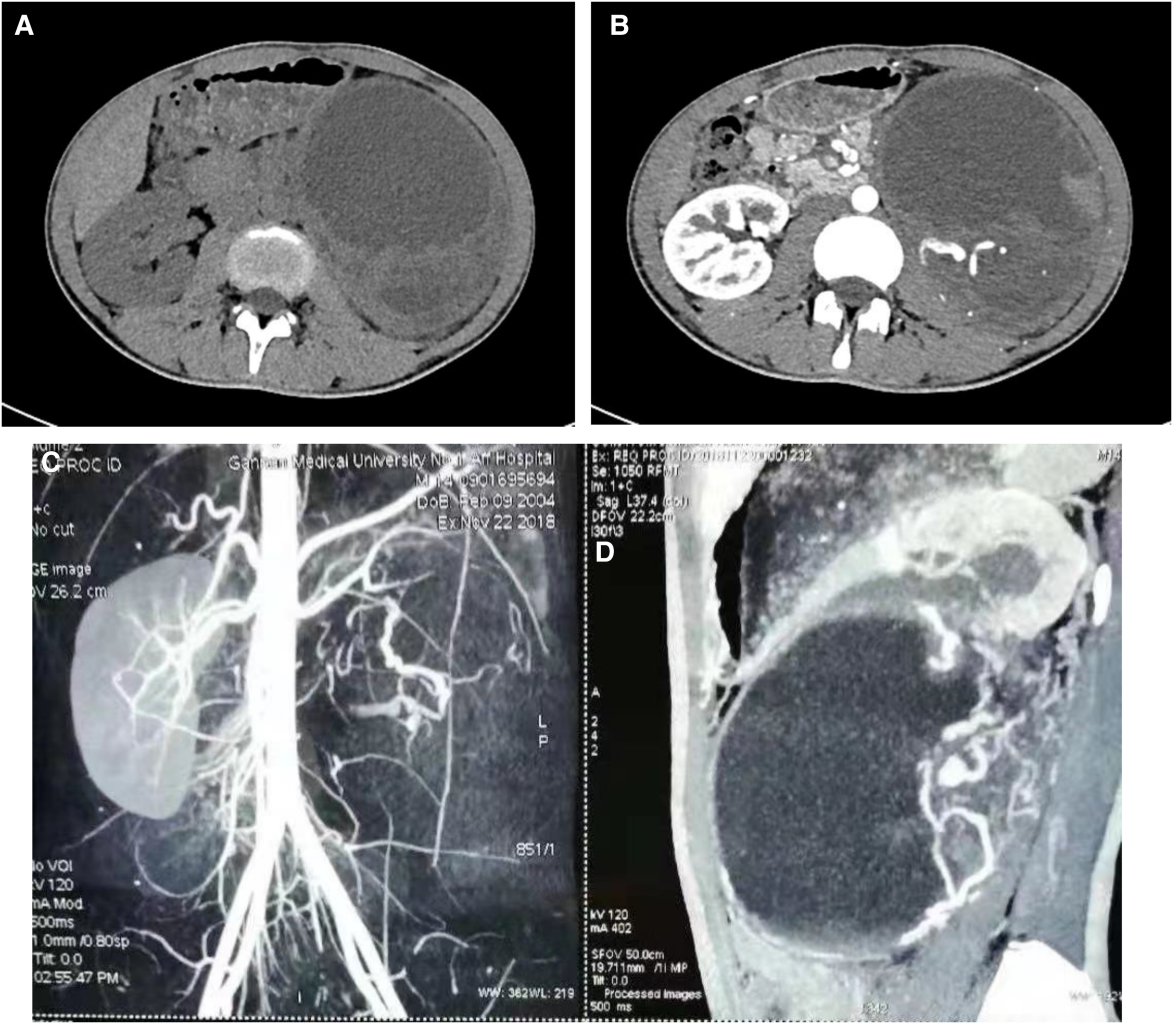
Ct scan of the urinary tract. CT scan of the venous phase of the left renal tumor (**A**), enhanced CT scan of the arterial phase of the left renal tumor (**B**), angiography of a tumor in the left kidney (**C**). Sagittal view of the mass (**D**).

**Figure 2:**
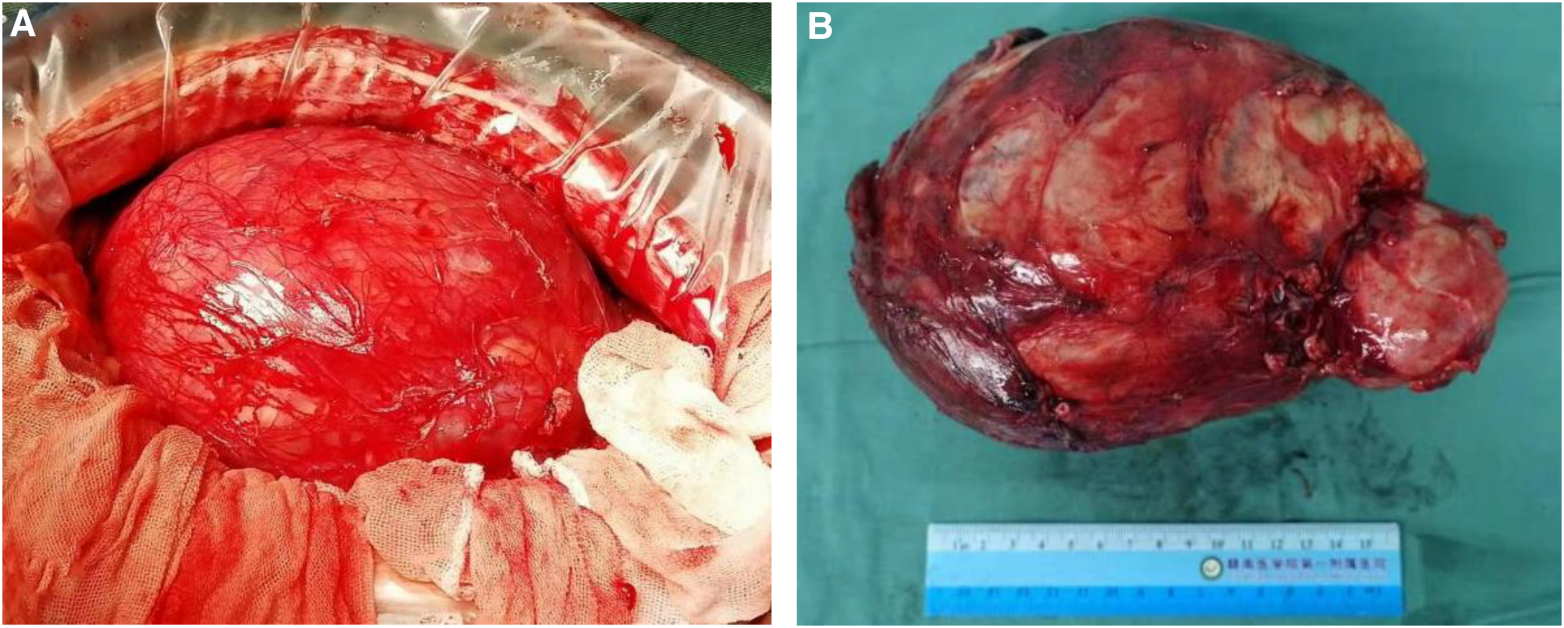
A radical resection of the left kidney was performed, and gross specimens of the tumor were obtained. The tumor was located at the lower pole of the left kidney with clear boundaries and no adhesion (**A**), large tumor in the lower pole of the left kidney, and the surface is smooth and locally cystic (**B**).

**Figure 3:**
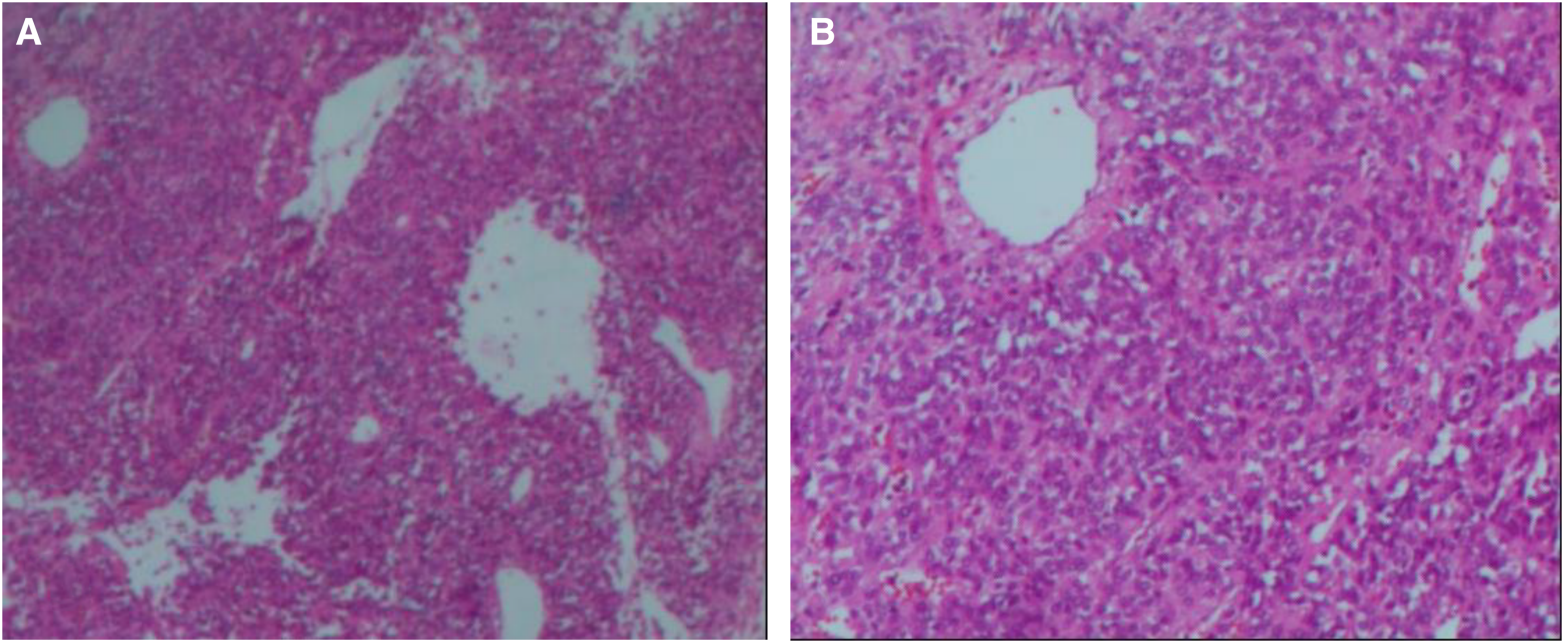
Microscopic appearance (**A,B**). Histological magnification revealed that the tumor cells are small and round [hematoxylin and eosin staining, × 200 (**A,B**)].

A 14-year-old male patient was admitted to the hospital with left-sided lumbago and abdominal pain, a 1-week fever, and no relevant medical history. At physical examination, a large mass could be felt under the costal margin of the left waist, which was hard in quality and fixed in position without tenderness or pulsation. The lower end of the umbilicus was flat, and there were no other obvious abnormalities. Auxiliary examination through a color ultrasound of the urinary system showed a mixed echo (13.2 cm × 10.9 cm) of the lower pole of the left kidney, of unknown nature and a clear border. Urinary CTU + CTA revealed a large mass of soft tissue density shadow (13.2 cm × 10.6 cm) in the lower pole of the left kidney. The mass showed uneven enhancement, but there was no enhancement in cystic lesion or necrotic area. The mass had coarse supplying arteries, and the CT value ranged from approximately 22 to 44 HU (Figures 1A–D). A chest CT showed no significant abnormalities. Two kidney ECTs were performed: left kidney GFR 11.38 ml/min, right kidney GFR 99.8 ml/min. There were no obvious anomalies in the whole-body bone MRI. Blood analysis and biochemical examination also showed no abnormalities. The preoperative diagnosis was a tumor of the left kidney. A radical nephrectomy was performed under general anesthesia. An intraoperative mass surrounding the left kidney of approximately 13 cm long was found in the organ's lower pole (Figure 2A). Postoperative gross specimens showed that the tissue size of the left kidney was 17.0 cm × 11.0 cm × 4.0 cm, with a smooth surface (Figure 2B), and had a grayish-yellow, polycystic, and mucous mass that measured 8.0 cm × 5.0 cm × 4.0 cm in the capsule. Microscopically, the tumor cells were small, round, and distributed in sheets, loaves, or strips (Figures 3A,B). Immunohistochemical results showed Vim (+), CD99 (+), NSE (+), CD56 (−), Ki-67 (20%+), Syn (−), CgA (−), S-100 (−). The postoperative pathological diagnosis was PNET of the left kidney. The patient recovered well after surgery and was discharged from the hospital. The patient was followed up for nearly 5 years, and no chemotherapy or radiotherapy was given; no tumor recurrence occurred.

## Discussion

PNET is a highly malignant small-round-cell tumor with neurogenic differentiation. PNET occurs more rarely in the kidney, although its clinical manifestations are similar to those of renal cell carcinoma: abdominal pain or lumbago, hematuria, fever, night sweats, and so on, even though the primary symptoms were the presence of an abdominal mass and weight loss. Risi (3) reported that 54% of patients presented with pain, 28% with a bulky renal mass, and 29% with hematuria. Compared to other renal tumors, rPNET malignancy is more common, prone to recurrence after surgery, and more likely to metastasize, with the most common sites of metastasis being the lungs, followed by the liver and bones (4). There are also early renal vein or inferior vena cava cancer thrombus. Ladenstein (5) showed that, as compared with patients with metastatic rPNET, patients without metastatic rPNET had a considerably higher 5-year survival rate after surgical treatment and subsequent chemotherapy. A total of 70% of patients in their study did not develop metastases, while 30% did. In conclusion, early metastasis or postoperative recurrence and metastasis are the most important reasons for the poor postoperative prognosis of patients.

The size of PNETs varies. Clinically, all rPNET tumor bodies are large, with a maximum diameter of 12.5 cm and above (1). According to Risi (3), the tumor size of rPNETs ranged from 3.3 to 18 cm, with a median of 13 cm. rPNETs are mainly flaky or lobular, soft, and often accompanied by necrosis and bleeding. Histologic features are small, uniformly round, or oval cells with most mitotic figures. rPNET cells can form neurofibrillary matrix cores and Homer-Wright rosettes, which are the most obvious diagnostic histological features of renal PNET. With the improvement of immunohistochemical techniques, they have become an important basis for the pathological diagnosis of rPNET (6). CD99 is a monoclonal antibody that recognizes p30/32 glycoprotein and contributes to the identification of rPNET cells (7). CD99 is detected in almost all rPNET (8). However, it has also been reported that CD99 is expressed in small-cell carcinoma, Wilms’ blastoma, and non-Hodgkin lymphoma (9). In addition, rPNET has a number of neural markers, including NSE, Leu-7, S-100 protein, synaptophysin, and pheochromoin A. It has been reported that the expression of CD99 in rPNET is as high as 90%, the expression of NSE in neural markers is 80%–90%, and the rest are below 50% (10). It is generally believed that the diagnosis of rPNET should conform to (1) Chrysanthemum-shaped clusters being visible under a light microscope; (2) CD99 and other neural markers being positive (at least two) (11). There is little correlation between prognosis and tumor size and morphology.

In recent years, reports on the molecular genetics of rPNET have been increasing, with more than 90% of these predicting that translocation of t (11; 22) (q24; q12) will occur. The EWS gene is found on chromosome 22q12 and has 17 exons, with most breakpoints occurring in exon 7. The FLI-1 gene found on chromosome 11q24 belongs to the ETS proto-oncogene family and acts as a transcriptional activating factor. Further research showed that the t (11; 22) (q24; q12) translocation leads to an EWS/FLI-1 radical fusion (12). The RNA binding region of a gene fuses with a transcription factor gene to form a new fusion gene in the fusion mode. The fusion of exon 7 with exon 6 is known as EWS/FLI-1 fusion, and exon 8 fusion is known as EWS/FLI-2 fusion. The fusion gene is located in the nucleus and is a more potent transcriptional activator than FL-1. Other ETS family members (e.g., ERG11) rarely fuse with EWS genes. According to de Alava (13), the prognosis of EWS/FLI-1 fusion was superior to that of EWS/FLI-2, and their study also found that the fusion genotype t (11; 22) was associated with tumor prognosis, with EWS/FLI-1 positive prognosis being better in cases without metastasis. Unfortunately, there is no technical molecular genetics monitoring at our facility.

At present, there is no unified standard for the treatment of rPNET, which mainly includes surgical treatment, chemotherapy, and radiotherapy. Surgical excision is mainly applicable to cases without metastasis, and radical nephrectomy is usually adopted. There have also been reported cases of nephrectomy with nephron-sparing partial nephrectomy and postoperative adjuvant chemotherapy, but no metastasis was found after 2 years of follow-up (14). It has also been reported that neoadjuvant chemotherapy can not only eliminate subclinical metastasis of the tumor but also facilitate surgical resection and reduce the volume of the primary tumor (15). Of course, there are also studies suggesting that neoadjuvant chemotherapy is not suitable for rPNET due to the difficulty of a preoperative diagnosis (16). When postoperative imaging or pathology indicates a positive margin or recurrence, radiotherapy may be chosen (17). In addition, new molecularly targeted therapies for ES/PNET are now moving from the laboratory to the clinical stage. Figitumumab, a human-like IgG2 monoclonal antibody against pancreatic growth-factor-1 receptors, showed encouraging results in patients with PNET in a recent phase I trial (18).

The prognosis of rPNET is generally poor, with an overall 5-year survival rate of 45%–55% (19). Thyavihally (10) reported a median survival time of 40 months, and 3-year and 5-year survival rates of 60% and 42%, respectively. Seth (20) reported a median survival time of 45 months, with 3-year and 5-year survival rates of 66% and 44%, respectively. In this case, no metastatic lesion was found in renal PNET prior to surgery, no neoadjuvant chemotherapy was performed, and only a radical nephrectomy was performed. The patient gave up postoperative radiotherapy and chemotherapy in order to avoid the effects of radiotherapy and chemotherapy on intellectual ability and its effects on academic performance. Postoperative radiotherapy, and chemotherapy were not administered in order to avoid the effects of radiotherapy and chemotherapy on intellectual ability and their effects on academic performance. Although there was no typical chrysanthemum mass structure in the postoperative pathology, the lump was confirmed as rPNET by immunohistochemistry. No recurrence or metastasis were found over the approximately 5-year follow-up period, during which we reviewed chest and whole abdomen CT every six months. We will continue to monitor the patient and expect a better prognosis.

## Conclusions

Despite the high prevalence of rPNET malignancy, patients without metastasis before surgery can still obtain a good survival prognosis through timely radical resection.

## Data Availability

The original contributions presented in the study are included in the article/Supplementary Material, further inquiries can be directed to the corresponding author.
